# Automatic image-based tracking of gadolinium-filled balloon wedge catheters for MRI-guided cardiac catheterization using deep learning

**DOI:** 10.3389/fcvm.2023.1233093

**Published:** 2023-09-07

**Authors:** Alexander Paul Neofytou, Grzegorz Tomasz Kowalik, Rohini Vidya Shankar, Li Huang, Tracy Moon, Nina Mellor, Reza Razavi, Radhouene Neji, Kuberan Pushparajah, Sébastien Roujol

**Affiliations:** ^1^ School of Biomedical Engineering and Imaging Sciences, Faculty of Life Sciences and Medicine, King’s College London, London, United Kingdom; ^2^ Department of Paediatric Cardiology, Evelina London Children's Hospital, London, United Kingdom; ^3^ MR Research Collaborations, Siemens Healthcare Limited, Camberley, United Kingdom

**Keywords:** MRI-guidance, cardiac catheterization, AI, device tracking, real-time

## Abstract

**Introduction:**

Magnetic Resonance Imaging (MRI) is a promising alternative to standard x-ray fluoroscopy for the guidance of cardiac catheterization procedures as it enables soft tissue visualization, avoids ionizing radiation and provides improved hemodynamic data. MRI-guided cardiac catheterization procedures currently require frequent manual tracking of the imaging plane during navigation to follow the tip of a gadolinium-filled balloon wedge catheter, which unnecessarily prolongs and complicates the procedures. Therefore, real-time automatic image-based detection of the catheter balloon has the potential to improve catheter visualization and navigation through automatic slice tracking.

**Methods:**

In this study, an automatic, parameter-free, deep-learning-based post-processing pipeline was developed for real-time detection of the catheter balloon. A U-Net architecture with a ResNet-34 encoder was trained on semi-artificial images for the segmentation of the catheter balloon. Post-processing steps were implemented to guarantee a unique estimate of the catheter tip coordinates. This approach was evaluated retrospectively in 7 patients (6M and 1F, age = 7 ± 5 year) who underwent an MRI-guided right heart catheterization procedure with all images acquired in an orientation unseen during training.

**Results:**

The overall accuracy, specificity and sensitivity of the proposed catheter tracking strategy over all 7 patients were 98.4 ± 2.0%, 99.9 ± 0.2% and 95.4 ± 5.5%, respectively. The computation time of the deep-learning-based segmentation step was ∼10 ms/image, indicating its compatibility with real-time constraints.

**Conclusion:**

Deep-learning-based catheter balloon tracking is feasible, accurate, parameter-free, and compatible with real-time conditions. Online integration of the technique and its evaluation in a larger patient cohort are now warranted to determine its benefit during MRI-guided cardiac catheterization.

## 1. Introduction

Cardiac catheterization is a common procedure used to diagnose and treat patients with a variety of conditions such as congenital heart disease (CHD) (1–3). Catheterization procedures are traditionally navigated under fluoroscopic guidance (4), which exposes patients to harmful ionizing radiation and the unwanted risk of developing cancer, particularly in children and young adults who undergo long and repeated procedures (5–7). Fluoroscopic guidance also suffers from poor soft tissue visualization, making it difficult to position catheters and balloons, with the potential increased risk of instrumental perforations in patients (5).

Magnetic Resonance Imaging (MRI)-guidance is an attractive alternative to fluoroscopic guidance as it involves no radiation risks to patients involved, has superior soft tissue visualization and superior hemodynamic data using MR flow imaging (8–18). MRI-compatible balloon-wedge catheters are used during these procedures. The catheter tip is visually tracked passively using dynamic real-time imaging (∼5–10 images/s) from its corresponding hyper- or hypo-intense signal when using diluted gadolinium or CO_2_-filled balloons, respectively (18). Several techniques exist for improved positive contrast visualization of the catheter tip when using gadolinium-filled balloons. These include the application of saturation pre-pulses intermittently (i.e., switching between balloon or blood/cardiac anatomy visualization) (18) or using black-blood preparation for simultaneous balloon and cardiac anatomy visualization (19). The use of partial saturation (pSAT) pre-pulses (i.e., a non-selective saturation pulse with a reduced flip angle, typically 30–70°) (16) has the advantage over the other two methods of providing simultaneous high-contrast visualization of the catheter tip, soft tissue and blood (17). Despite the benefits of MRI-guidance, several major technical challenges remain to be addressed to ensure clinical acceptance of this method. Current imaging techniques require frequent manual updating of the imaging plane location to track the catheter tip during navigation to deal with the catheter tip frequently falling out-of-plane. This typically arises when navigating the catheter within cardiac chambers, large vessels, or complex anatomical vessels which cannot be fully sampled using a 2D imaging plane. One study reported an average balloon visibility of <70% of the scanning time (17). This limitation leads to the unnecessary complication and slowdown of catheterization procedures when relocating the catheter tip. It has been shown that by acquiring a heavily T1-weighted image of the catheter using a large slice thickness and overlaying this onto a high-resolution image of the anatomy of interest improves catheter visibility to ∼90% of the scanning time (20). However, the larger slice thickness may also reduce the value of MRI-guidance in narrow anatomical structures.

Automatic tracking of the catheter and imaging plane during navigation may potentially facilitate and shorten the procedure. In a recent proof-of-concept study, automatic slice tracking based on real-time image processing and estimation of the catheter tip location was proposed to enable continuous visualization of the catheter tip during navigation (21). This approach relies on the dynamic acquisition of three contiguous slices, typically using a bSSFP readout for improved signal-to-noise ratio and reduced flow artefacts. When the catheter is detected in one of the outer slices, the image plane is prospectively adjusted automatically to maintain the catheter in the central slice. Despite the potential of this framework to achieve near 100% visibility of the catheter during navigation, it depends on several user-definable parameters, such as a bounding box definition to contain expected catheter trajectories, image segmentation threshold values, pattern matching and catheter movement speed. These parameters not only rely on user experience but may reduce the framework's robustness in patients.

In this study, an automatic, parameter-free, deep-learning-based post-processing pipeline was developed for robust real-time image-based detection of the catheter tip coordinates. This method was evaluated retrospectively in seven patients who underwent MRI-guided right heart catheterization using the aforementioned prototype catheter tracking sequence.

## 2. Methods

All imaging was performed on a 1.5 T MRI scanner (MAGNETOM Aera, Siemens Healthineers, Erlangen, Germany).

### 2.1. Proposed model architecture

For semantic segmentation (i.e., pixel-wise classification) of the catheter balloon, the commonly used U-Net architecture (22) was employed for both its high-resolution localization of objects and global context understanding, owing to its encoder-decoder structure. Here a modified U-Net model using a ResNet-34 (23) encoder (i.e., a 34 layered Residual Neural Network) was employed as it was shown to provide higher recall when compared to the standard U-Net architecture with imbalanced datasets (24), as is the case in this study (i.e., the balloon represents <1% of the image). The U-Net consisted of pre-trained weights using the ImageNet dataset (25). The input to the network is a magnitude image and the output is a binary mask containing the segmented catheter balloon signal. The sigmoid activation function in the final layer was implemented to predict the class of an underlying pixel, with an activation value greater than or equal to 0.5 being classed as balloon signal and less than 0.5 being classed as otherwise (i.e., background).

### 2.2. Training data

The proposed network was trained using semi-artificial data consisting of images with retrospectively placed artificial catheter balloon signal, as follows. Twelve patients (8 male and 4 female, age = 40 ± 15 year) undergoing a routine clinical cardiac magnetic resonance examination (i.e., no cardiac catheterization procedure performed) were recruited to generate training (8 patients) and validation (4 patients) data for this study, which was approved by the National Research Ethics Service (REC reference: 15/NS/0030). Written informed consent was obtained from all patients for the scan and for inclusion in this study. For each patient, a stack of 20 slices in the transverse and coronal orientations was acquired (i.e., 40 slices/patient) with a pSAT prepulse (pSAT angle = 40°) and conventional real-time bSSFP imaging. Note that none of the training data was acquired in the sagittal orientation to evaluate the potential of the network to operate on an existing validation dataset composed of an unseen orientation (i.e., only the sagittal orientation. See Section 2.4 *in-vivo* evaluation in MRI-guided cardiac catheterization patient data). The following imaging parameters were used: TE/TR = 1.25/2.5 ms, Flip angle = 70°, FOV = 400 × 400 mm^2^, reconstructed/acquired resolution = 1.6 × 1.6/3.3 × 3.1 mm^2^, slice thickness = 10 mm, BW = 1002 Hz/pixel, GRAPPA factor = 2.

Artificial catheter balloon signal was generated in these images, blinded from the testing data acquired during clinical MRI-guided cardiac procedures and described in Section 2.4. The catheter balloon was simulated in locations of the cardiovascular anatomy commonly navigated during cardiac catheterization procedures. For each image depicting the cardiovascular system, multiple realistic catheter positions were manually generated. One simulated training image was created for each manually defined catheter location since only one catheter can be present at a time in these procedures. At each predefined location, the catheter shape was emulated as a 2D anisotropic Gaussian. To vary the shape, size and signal amplitude of the artificial catheter signal typically observed across patients, the 2D Gaussian model at each point was generated with:

1. A randomly selected standard deviation value of either 1.5 or 2.5 pixels (i.e., $\sigma_{x}\;=\{1.5,\;2.5\}$ and $\sigma_{y}=\{1.5,\;2.5\}$).
2. A randomly selected integer rotation of the catheter balloon in the range of 0°–360° (step size = 1°).
3. A randomly selected Gaussian signal amplitude (range: 200%–400%, step size = 100%) of the underlying signal intensity (i.e., at the pre-selected central point of the Gaussian).

**Figure 1:**
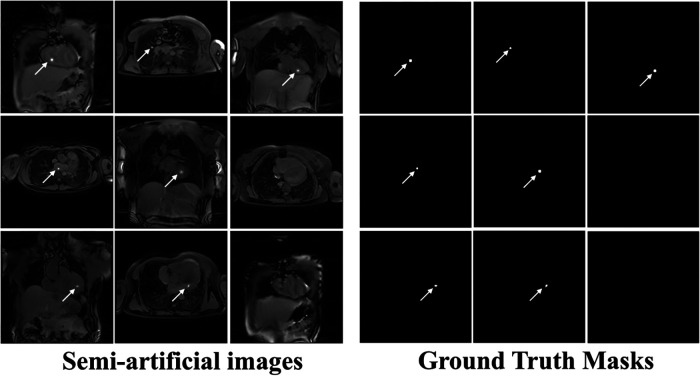
Example “semi-artificial” images containing retrospectively placed catheter signal and their respective ground truth segmentation masks. Catheter balloon signal modelled as a 2D anisotropic Gaussian with standard deviation ($\sigma_{x}$ and $\sigma_{y}$) ranging from 1.5 to 2.5 pixels (step size = 1 pixel). Various intensities ranging from 200 to 400% (step size = 100*%*) of the underlying signal intensity (i.e., at the central pixel of the Gaussian) were used. Random balloon rotations ranging from 0° to 360° (step size = 1°) were used. The respective ground truth masks, segmenting the artificial balloon signal were generated by setting all pixels within 1.5 standard deviations of the Gaussian profile as 1 and 0 elsewhere.

Overall, a total of 4,744 (3,269 training and 1,475 validation) unique semi-artificial images were generated from the original 480 image dataset (i.e., ∼10-fold increase) for the training of the proposed network (refer to Figure 1 for representative examples). Note, 10% of the training data included images with no simulated catheter balloon to help the network minimize false positive detection.

Respective ground truth masks were automatically generated by selecting all pixels located within the mean ± 1.5 × standard deviation of the Gaussian distribution profile used for each simulated catheter balloon signal (see Figure 1).

### 2.3. Model training

There was a severe class imbalance between “balloon” class pixels relative to “background” pixels in the segmentation masks, with “background” pixels representing ∼99% of the total pixels. For this reason the Dice loss function was implemented for its robustness in dealing with unbalanced classes (26). The training and validation dice loss plots are shown in Supplementary Figure S1.

Training occurred over 200 epochs, with a learning rate of 1e−5 and batch size of 64 images using the adaptive moment estimation (ADAM) optimizer. The epoch where the validation loss function was minimized (i.e., the global minimum) was chosen and the corresponding set of model weights were used for the final trained model. Training and testing of the network were performed using a SCAN workstation (AMD Ryzen 9 5950X 16-Core Processor running at 3.40 GHz, 128 GB of RAM, NVIDIA RTX A6000 GPU). The PyTorch library for deep learning was used.

### 2.4. *In-vivo* evaluation in MRI-guided cardiac catheterization patient data

De-identified data from seven patients (6M and 1F, age = 7 ± 5 year) who underwent MRI-guided right heart catheterization were used for retrospective evaluation of the proposed approach, with approval from our local institutional review board via waiver of informed consent (REC reference: 21/LO/0650). A prototype catheter tracking sequence was run to generate three contiguous slices, acquired dynamically using a bSSFP readout and the following parameters: TE/TR = 0.99/2.44 ms, Flip angle = 50°, FOV = 450 × 450 mm^2^, reconstructed/acquired resolution = 1.4 × 1.4/2.8 × 2.8 mm^2^, slice thickness = 10 mm, number of dynamic measurements = 8–116, bandwidth = 1,010 Hz/px, GRAPPA factor = 2, partial Fourier = 5/8, pSAT angle = 30–70°. This sequence was run for a short period during the catheterization procedure. The balloon of a 4–6 French wedge catheter was filled with 1% Gadolinium (Dotarem®, Guerbet, Villepinte, France) for positive contrast visualization. A total of 858 images were acquired over the seven patients, all in the sagittal orientation.

The following steps of the proposed post-processing strategy were applied independently for each dynamic (i.e., set of three slices per dynamic):

1. Using the trained neural network, generate a segmentation mask for each of the three contiguous slices.
2. To remove any ambiguities if multiple catheter balloon signal were identified across and/or within the three slices, the region with the highest signal intensity was selected as the final segmentation of the catheter balloon across the three slices. This can occasionally happen if the catheter is visible across two contiguous slices. In this case, it is important to determine the slice containing most of the catheter (i.e. higher signal intensity) to enable appropriate prospective slice tracking.

All images (i.e., training, validation and testing) were normalized by first scaling in the range 0–1, then subtracting by the mean pixel value and dividing by the standard deviation pixel value of the ImageNet dataset.

### 2.5. Model evaluation

Ground truth segmentation of the catheter balloon was first manually and independently defined in each slice and dynamic where the catheter was deemed visible. This was performed by AN by drawing a circular ROI over the catheter balloon and generating a ground truth (GT) binary mask in MATLAB (MathWorks, Natick MA, USA). This enabled the assessment of accuracy, specificity and sensitivity of the network alone in detecting the presence/lack of catheter signal on a per-image basis.

The accuracy, specificity and sensitivity of the complete post-processing strategy (which outputs the best candidate region across all slices) were then evaluated. To this end, a multi-slice ground truth was generated by only keeping the manually segmented ground truth region across the three slices with the highest signal intensity.

For both analyses, a correct detection [i.e., true positive (TP)] was considered only for a single segmented region and its center of mass intersecting the ground truth region of the balloon signal, else classified as an incorrect detection [i.e., false positive (FP)]. A correct “no detection” was defined as an absence of catheter segmentation on both prediction and ground truth, which was classified as a true negative (TN). If the network completely failed to output a mask that segments the true catheter signal this was classified as a false negative (FN). Please refer to Supplementary Figure S2 for illustrative examples. Accuracy, sensitivity and specificity were measured independently for each patient. A mean accuracy, sensitivity and specificity was then calculated as the average over all patients of the patient-specific accuracy, sensitivity and specificity, respectively.

Inter- and intra-operator variability on the segmentation of the catheter balloon was also performed on a sub-set of 70 randomly selected images (10 per patient). The segmentation of the catheter balloon was repeated by AN and KP independently in order to assess intra- and inter-operator variability. Agreement between two segmentations was defined as the center of mass of each of the two masks falling inside its corresponding one. The percentage of agreement between segmentations is reported as a measure of intra- and inter-operator variability.

## 3. Results

Supplementary Figure S1 shows the training and validation dice loss plot over 200 epochs. The global minimum of the validation loss was determined at epoch 169.

**Figure 2:**
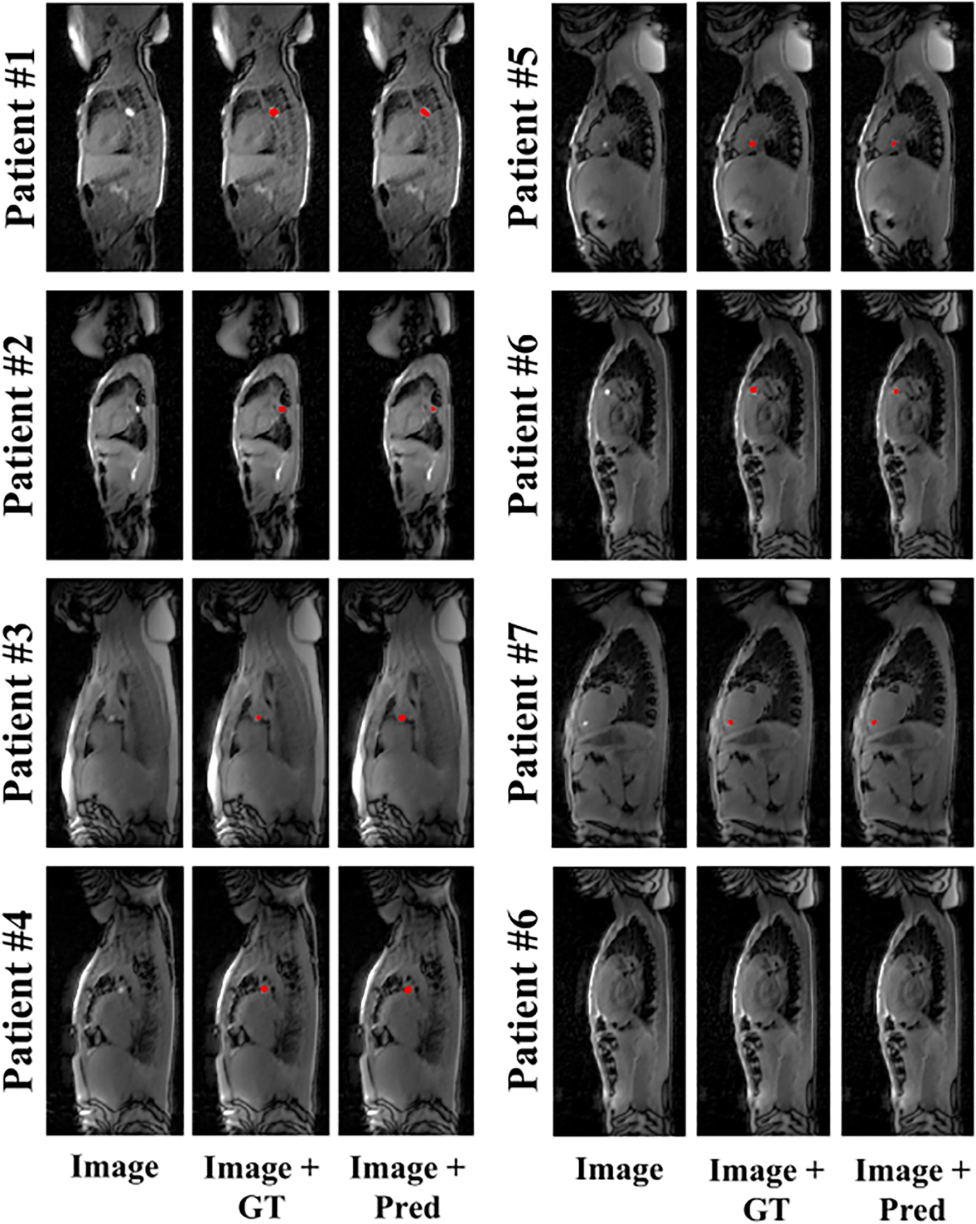
Representative example predictions of the network for all patients who underwent MRI-guided cardiac catheterization. Image (input to the network), ground truth (GT) segmentation masks overlaid onto the input image (i.e., Image + GT) and network prediction masks (Pred) overlaid onto the input images (i.e., Image + Pred) are shown for each subject. Accurate catheter segmentation was achieved for each of the seven patients. An additional true negative example (patient #6) is provided (bottom right).

Representative example predictions of the network alone, for each of the seven patients who underwent MRI-guided cardiac catheterization, are shown in Figure 2. A large variation of catheter-to-blood contrast, catheter balloon size/shape, and overall anatomy can be observed within this patient cohort. Despite these variations, accurate prediction of the catheter balloon was achieved in all subjects using the proposed network. One representative example of true negative detection is also shown (see the bottom right case in Figure 2). No catheter was present in any part of this image. Interestingly, despite the presence of hyper-intense signal, specifically from fatty tissue, the network was able to predict the absence of the catheter balloon. On an image basis, the mean accuracy, sensitivity and specificity of the network alone over all patients were 87.5 ± 10.9%, 93.4 ± 13.8% and 85.3 ± 13.8%, respectively.

**Figure 3:**
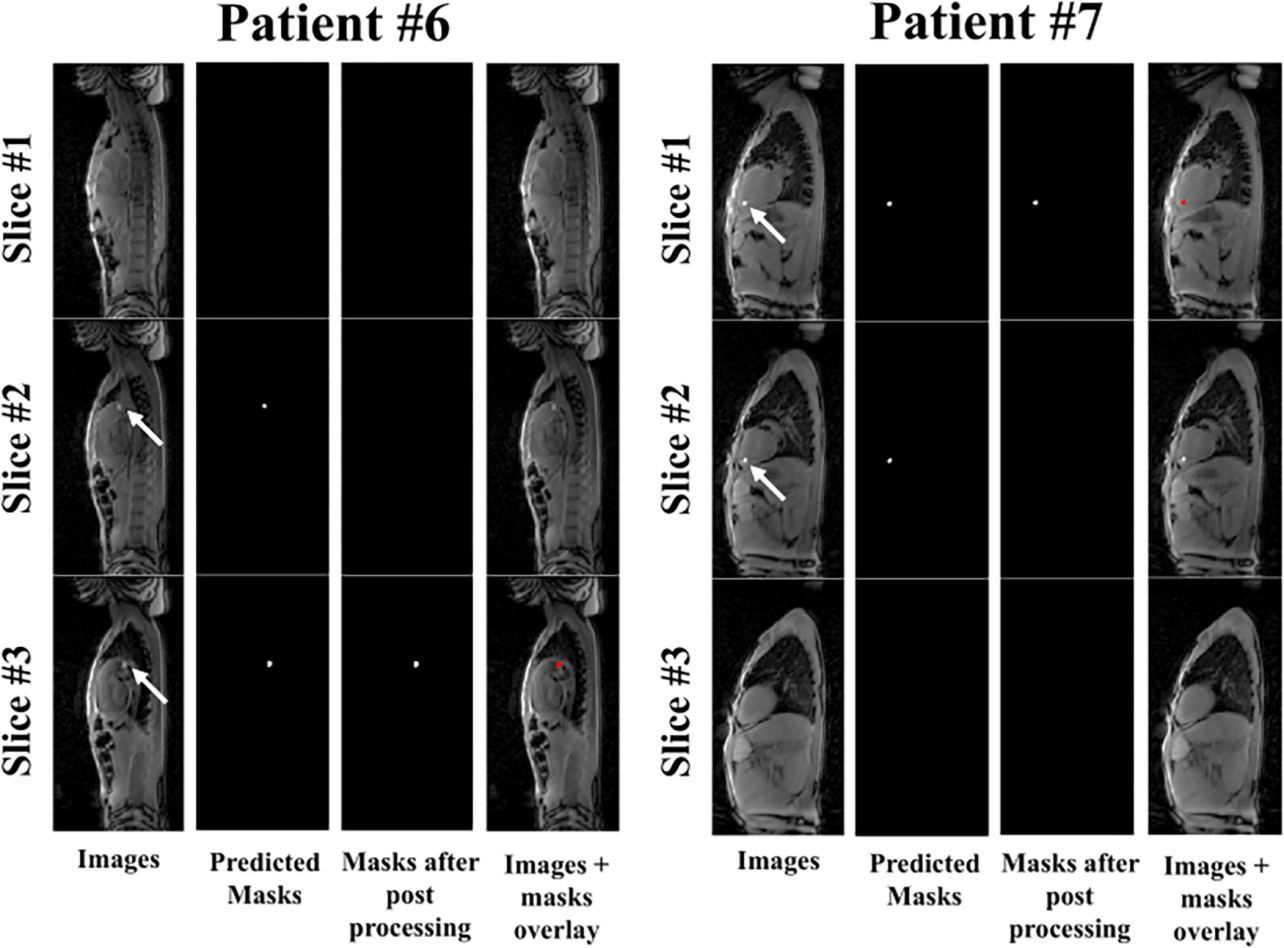
The complete reconstruction pipeline shown for patients #6 and #7. Here the tracking of the catheter is depicted in the three dynamically acquired contiguous images. The white arrows point to the location of the balloon within a given slice. Please refer to Supplementary Video S1 for the corresponding dynamic video for patient #6. Accurate catheter tracking was achieved in most dynamics across the three contiguous slices.

Figure 3 depicts the complete reconstruction pipeline in two patient examples (patients #6 and #7) where the tracking of the catheter is depicted in the three dynamically acquired contiguous images. Significant bright fat tissue is also visible in these images but did not affect the output of the network. Furthermore, the catheter was initially detected in two adjacent slices for both patients using the network alone. The proposed post-processing strategy resolves this ambiguity and identifies the slice containing most of the catheter. Supplementary Video S1 shows the corresponding dynamic video for patient #6. Accurate catheter detection was achieved in the majority of dynamics.

The detailed accuracy, specificity and sensitivity measured for each patient are shown in Table 1. While the worst accuracy was 95.2% in patient #4, an accuracy of 100% was achieved in 4/7 patients. Over all patients, the mean accuracy, specificity and sensitivity of the complete catheter tracking pipeline were 98.4 ± 2.0%, 99.9 ± 0.2% and 95.4 ± 5.5%, respectively.

**Table 1:** Accuracy, specificity and sensitivity (%) values for each patient for the complete catheter tracking pipeline.

Patient	Accuracy (%)	Specificity (%)	Sensitivity (%)
1	100.0	100.0	100.0
2	100.0	100.0	100.0
3	100.0	100.0	100.0
4	95.2	100.0	86.4
5	100.0	100.0	100.0
6	97.3	99.3	92.6
7	96.3	100.0	89.0

The mean (measured across all patients) accuracy, specificity and sensitivity are 98.4 ± 2.0%, 99.9 ± 0.2% and 95.4 ± 5.5%, respectively.

High repeatability and reproducibility of the manual segmentation of the catheter was observed, as denoted by an intra- and inter-operator percentage of agreement of 100% and 100%, respectively.

The computational time of this automatic tracking pipeline was ∼30 ms, with the network prediction accounting for ∼10 ms/image.

## 4. Discussion

In this work, an automated, parameter-free, post-processing strategy was developed using deep-learning for image-based detection of gadolinium-filled balloon wedge catheters during cardiac catheterization procedures. The proposed method provides excellent accuracy, sensitivity and specificity, with computation times compatible with real-time constraints.

Semi-artificial data were used for the training process due to the limited availability of real data. The fact that semi-artificial data can be successfully used for the training of such a network is an important finding, especially when the scarcity of very large training datasets for artificial intelligence applications has been identified as a major obstacle, particularly in the field of cardiac magnetic resonance imaging (27). This also greatly reduces the time-consuming and laborious nature of manually annotating large volumes of data required for training. A potential limitation to placing artificial catheter signal on underlying images is that signal may not fully represent the variability (i.e., shape and intensity) observed in real-world data. Similarly, the choice made by a single operator of placing the artificial signal at specific regions in the cardiovascular anatomy could lead to bias in the training data. However, the high performance of the proposed approach observed in this study indicates these effects may be limited.

Furthermore, all semi-artificial data were based on images acquired in adults and two orientations only (transverse and coronal). The test patient cohort consisted predominately of paediatric patients with all images acquired in an unseen orientation during the training (i.e., sagittal). Imaging protocols used for the acquisition of the training and testing datasets also had slightly different imaging parameters. Finally, images from this test cohort had high variability in terms of anatomy as well as catheter balloon contrast/shape. Therefore, the high accuracy of the proposed tracking strategy in the test dataset suggests that the network was able to adapt to different scenarios unseen during the training process. The network adaptability is particularly important since large anatomical variations are expected in the patient population referred for MRI-guided cardiac catheterization and different slice orientations are also expected to be used during catheter navigation (17).

The simplicity and success of the U-net model and its variants have resulted in its extensive adoption as the primary tool for segmentation tasks within the medical imaging community (22, 28–31) and thus was the choice of network used in this study. As discussed in Section 2.1., the U-Net model was used incorporating a ResNet-34 encoder. This model proved to be highly effective in achieving high accuracy, specificity, and sensitivity in the detection of the presence or absence of balloon signal. It is important to note that ResNet-34 is one of several potential choices for the encoder in the U-Net architecture. Other popular encoder architectures include, for example, VGG-19 (32), Inception (33) and SE-Net (34) which have shown to provide good performance for semantic segmentation in medical imaging (29, 35–37). Such models could be explored in future work to further improve the performance of the network. Future work using segmentation models that leverage the temporal dimension of data to improve segmentation accuracy could also be investigated, such as the use of convolutional LSTMs (12).

Gadolinium-filled balloon catheters were used in this study. Gas-filled balloon catheters can also be used and are visualized through the signal voids they generate. Although gas-filled balloons were shown to be less conspicuous than Gadolinium-filled balloons, they offer improved buoyancy which may be beneficial for catheter navigation (13). Retraining of the network using images with negative contrast is expected to be required and could be facilitated using the existing simulated dataset which enables easy generation of varying catheter contrasts. The potential of the proposed tracking strategy for negative contrast catheter will be the focus of future work.

Although the proposed tracking strategy was applied for the detection of gadolinium-filled balloon wedge catheters during MRI-guide cardiac catheterization procedures, it may also benefit the tracking of other interventional devices. For example, the network could be retrained to learn specific signal signatures produced by an MR-compatible guidewire or needle.

This study has some limitations. First, the catheter tracking sequence used in this study relies on the dynamic acquisition of three contiguous slices, which results in a 3-fold reduction of the temporal resolution. Future work will include the integration of advanced acceleration techniques to improve the temporal resolution of the framework. Second, this is a retrospective study in a small patient cohort demonstrating the potential of automatic parameter-free image-based tracking of the catheter balloon. A further prospective study, including the online integration of the proposed approach in a larger patient cohort during an entire cardiac catheterization procedure, is now needed to assess its clinical impact, both in terms of catheter tip visualization, and prospective tracking during catheter navigation.

In conclusion, deep-learning-based catheter balloon tracking is feasible, accurate, parameter-free and compatible with real-time conditions. Online integration of the technique and its evaluation in a larger patient cohort are now warranted to determine its benefit during MRI-guided cardiac catheterization.

## Data Availability

The raw data supporting the conclusions of this article will be made available by the authors, without undue reservation.
