# Supplementary material for: Automatic image-based tracking of gadolinium-filled balloon wedge catheters for MRI-guided cardiac catheterization using deep learning
Source: Front Cardiovasc Med. 2023 Sep 7;10:1233093. doi: 10.3389/fcvm.2023.1233093 (PMC10513169; doi:10.3389/fcvm.2023.1233093)
Supplement: Supplementary file 2 [file Datasheet1.docx]

Supplementary Material

Automatic image-based tracking of gadolinium-filled balloon wedge catheters for MRI-guided cardiac catheterization using deep learning

Alexander Paul Neofytou^1^, Grzegorz Tomasz Kowalik^1^, Rohini Vidya Shankar ^1^, Li Huang^1^, Tracy Moon^2^, Nina Mellor^2^, Reza Razavi^1^, Radhouene Neji^1,3^, Kuberan Pushparajah^1^, Sébastien Roujol^1^*

*** Correspondance:** Dr. Sébastien Roujol: sebastien.roujol@kcl.ac.uk

# Supplementary Figures

#
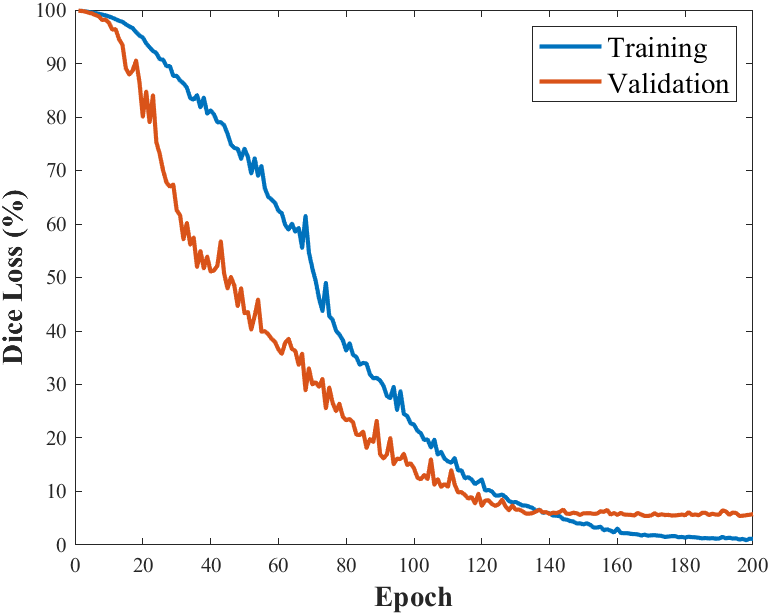


**Supplementary Figure S1.** Training and validation dice loss plots. The dice loss (validation) is minimized at the 169^th^ epoch.

**Supplementary Figure S2.** Example images illustrating [top left] a correct “no detection” (i.e., true negative (TN)), [top right] a failed detection of catheter signal (i.e., a false negative (FN)), [bottom left] an incorrect detection of catheter signal (i.e., a false positive (FP)) and a correct detection of catheter signal (i.e., a true positive (TP)).

# Supplementary Video

See Video 1.mp4 file.

**Supplementary Video S1.** Dynamic example depicting the complete reconstruction pipeline for patient 6. The white arrows point to the location of the balloon within a given slice.
